# Nicotinamide Inhibits CD4+ T-Cell Activation and Function

**DOI:** 10.3390/cells14080560

**Published:** 2025-04-08

**Authors:** Lotte Nijhuis, Alejandra Bodelόn, Rianne C. Scholman, Isabelle Houtzager, Lyanne J. P. M. Sijbers, Enric Mocholi, Lucas W. Picavet, Jorg J. A. Calis, Michal Mokry, Sebastiaan J. Vastert, Jorg van Loosdregt

**Affiliations:** 1 Center for Translational Immunology, University Medical Center Utrecht, Lundlaan 6, 3584 EA Utrecht, The Netherlands; l.nijhuis@umcutrecht.nl (L.N.); a.bodelondefrutos@umcutrecht.nl (A.B.); r.c.scholman@umcutrecht.nl (R.C.S.); l.j.p.m.sijbers@umcutrecht.nl (L.J.P.M.S.); b.vastert@umcutrecht.nl (S.J.V.); 2 Center for Molecular Medicine, University Medical Center Utrecht, Universiteitsweg 100, 3584 CG Utrecht, The Netherlands; emocholi@uchceu.es; 3 Department of Experimental Cardiology, University Medical Center Utrecht, 3584 CX Utrecht, The Netherlands; m.mokry@umcutrecht.nl; 4 Department of Pediatric Rheumatology and Immunology, University Medical Center Utrecht, Lundlaan 6, 3584 CX Utrecht, The Netherlands

**Keywords:** NAM, nicotinamide, CD4+ T-cell activation, auto-immune disease

## Abstract

Chronic inflammation and autoimmune diseases are driven, in part, by the activation of (auto)reactive CD4+ T-cells, highlighting their potential as therapeutic targets for these diseases. Nicotinamide (NAM) has demonstrated anti-inflammatory properties in various disease models and has already demonstrated safety in several large clinical trials in humans. The mechanisms behind these observations, and especially their direct effects on CD4+ T-cells, remain poorly understood. Here, we address this gap by investigating how NAM influences CD4+ T-cell activation and function. We also describe that NAM treatment significantly suppresses CD4+ T-cell activation in vitro, as evidenced by impaired proliferation and reduced expression of surface activation markers. Additionally, NAM treatment resulted in reduced production of pro-inflammatory cytokines, IL-2, IFNy, and IL-17, further highlighting its anti-inflammatory potential. We found that NAM modulates key metabolic processes, including glycolysis and reactive oxygen species (ROS) production—both essential to T-cell activation. Taken together, our findings provide novel mechanistic insight into the regulation of T-cell activation by NAM, suggesting NAM as an attractive candidate for novel therapies targeting immune-related diseases.

## 1. Introduction

T-cells are crucial players in the adaptive immune system, fulfilling a central role in orchestrating an effective and specific immune response against pathogens. CD8+ T-cells are primarily responsible for killing infected cells, while CD4+ T-cells coordinate and regulate immune responses by assisting other immune cells, such as B-cells and cytotoxic T-cells, through the release of cytokines [1]. While their function is critical for protecting the body from infections and malignancies, excessive activation of these T-cells can lead to direct tissue damage [2]. Increased activation of T-cells can promote the recruitment of additional immune cells, including macrophages, dendritic cells, and other T-cells, exacerbating the inflammatory response. This cascade is characteristic of autoimmune diseases such as rheumatoid arthritis, multiple sclerosis, and systemic lupus erythematosus [3,4,5]. As a result, CD4+ T-cells are considered critical drivers of autoimmune disease progression, and safely targeting CD4+ T-cell function is an important strategy in the treatment of autoimmune diseases [6,7].

One promising potential therapeutic approach to suppress immune activation in auto-immunity involves the use of Nicotinamide (NAM), also known as niacinamide. NAM is a form of the food additive vitamin B3 and has been recognized for its anti-inflammatory properties in various disease models for over 50 years [8,9,10,11]. Notably, NAM has been demonstrated to protect against axonal degeneration in autoimmune encephalitis models [10]. In Type 1 diabetes research, NAM has demonstrated the ability to prevent the induction of diabetes in rats [8], and oral administration in humans has been described to preserve beta-cell function and prevent the onset of clinical diabetes in Islet Cell Antibodies—positive first-degree relatives of type-1 diabetics [12]. In addition, NAM has been suggested to reduce insulin requirements in newly diagnosed children with diabetes [11]. Furthermore, in a study published in 1959, a small cohort of human patients with arthritis treated with NAM exhibited clinical improvement [9]. Despite these promising findings, the precise mechanisms underlying NAM’s anti-inflammatory effects in auto-immune diseases remain unclear and are a topic of ongoing debate.

NAM has been demonstrated to exert effects across different cell types, impacting various cellular pathways. For instance, in human endothelial cells, NAM treatment resulted in a reduced expression of IFNy-induced ICAM-1 and HLA-DR antigen [13]. NAM might also impair immune activation by inhibiting Poly (ADP-ribose) polymerase 1 (PARP-1) and the NAD+ dependent deacetylase Sirtuin 1 (SIRT-1). Both PARP-1 and SIRT-1 inhibition have been demonstrated to reduce the activation of different immune cells, including monocytes and T-cells [14,15,16,17,18,19]. Additionally, NAM has been reported to enhance regulatory T-cell (Treg) function by increasing the expression of the transcription factor FOXP3 [20] via SIRT1-inhibition. Although the mechanisms of how NAM affects T-cell function in autoimmune disease remain largely unknown, increasing evidence suggests that it can modulate CD8+ T-cell activity, promoting anti-inflammatory effects while inducing differentiation into T-effector cells [21,22]. However, the function of NAM on CD4+ T-cells remains unclear.

In this study, we explored the effect of NAM on CD4+ T-cell function to further understand its therapeutic potential in the context of autoimmune diseases. We demonstrate that NAM effectively inhibits CD4+ T-cell activation, as evidenced by reduced expression of surface activation markers, inhibition of T-cell proliferation, and decrease in pro-inflammatory cytokine secretion. Additionally, we show that NAM suppresses key metabolic processes involved in CD4+ T-cell activation, including glycolysis, glucose transport, and reactive oxygen species (ROS) production. These results underscore the anti-inflammatory effects of NAM on CD4+ T-cells. A deeper understanding of these mechanisms may provide further insights into NAM’s potential role as a promising, relatively safe, additional therapeutic approach for autoimmune disorders.

## 2. Materials and Methods

### 2.1. Cell Culture

Human peripheral blood mononuclear cells (PBMCs) were obtained from blood from healthy volunteers and isolated using Ficoll-paque plus density gradient media.

CD4+ T-cells were isolated from PBMC using automatic magnetic activated cell sorting (Miltenyi Biotech, Bergisch Gladbach, Germany) using the CD4+ T-cell negative selection isolation kit (Miltenyi Biotech, Bergisch Gladbach, Germany, catalog number 130-096-533) according to the manufacturers protocol. Cells were cultured in RPMI 1640 medium (Thermo Fisher, Hampton, NH, USA) supplemented with 10% heat-inactivated human AB serum (Merck Life Science, Darmstadt, Germany) in the presence of 1% glutamine (Merck Life Science) and 1% penicillin–streptomycin (Merck Life Science). For stimulation, cells were incubated in the presence of either anti-CD3/anti-CD28 Dynabeads (Thermo Fisher) in a ratio of 1:3 (bead/cells) unless indicated otherwise. Nicotinamide (Sigma) was added to the treatment condition at a concentration of 9 mM. All cells were cultured at 37 °C in 5% CO_2._ CD8+ T-cells were isolated from PBMC using the CD8+ T-cell negative selection isolation kit (Miltenyi Biotech, Bergisch Gladbach, Germany, catalog number 130-096-495) according to the manufacturer’s protocol. Following isolation, the cells were cultured using the conditions, as described for CD4+ T-cells.

### 2.2. Flow Cytometry

Cells were washed in FACS buffer (PBS with 2% FCS and 0.1% NaN_3_) and stained with antibodies (Supplementary Table S1). For intracellular staining, cells were fixed and treated with Golgistop (BDBioscience, Franklin Lakes, NJ, USA) in a 1:1500 dilution for the last 4 h of incubation. Measurements were performed using the BD FACSCanto™ II flow cytometer, and FlowJo v10 was used for data analysis.

### 2.3. qPCR

Cells were washed in PBS and lysed in RLT buffer (RNeasy kit, Qiagen, Hilden, Germany) and stored at −20 °C before RNA isolation. RNA was isolated using the manufacturer’s protocol. cDNA synthesis was performed using the iScript cDNA synthesis kit (Bio-Rad, Hercules, CA, USA). cDNA was stored at −20 °C. qPCR analysis was performed on the Quantstudio 3 real-time PCR system with SYBR select master mix (Thermo Fisher, Waltham, MA, USA) and the following primer sets: B2M forward primer TGCTGTCTCCATGTTTGATGTATCT, B2M reverse primer TCTCTGCTCCCCACCTCTAAGT; IL2 forward primer AACTCACCAGGATGCTCACATTTA, IL2 reverse primer TCCCTGGGTCTTAAGTGAAAGTTT; IFNγ forward primer GCAGAGCCAAATTGTCTCCT, IFNγ reverse primer ATGCTCTTCGACCTCGAAAC, TNF forward primer CCCCAGGGACCTCTCTCTAA, TNF reverse primer TGAGGTACAGGCCCTCTGAT, IL17α forward primer CCGTGGGCTGCACCTGTGTC, IL17α reverse primer GGGAGTGTGGGCTCCCCAGA; CD69 forward primer AGCTGGACTTCAGCCCAAAA, CD69 reverse primer ACCCTGTAACGTTGAACCAGT. Housekeeper for CD8+ T-cells; GUSB forward primer AGACAAGGGGGCTCCGTA, GUSB reverse primer CGTTTCTGCTCCATACTCGC

### 2.4. Luminex

Isolated CD4+ T-cells (1 × 10^6^) were activated with aCD3/aCD28 bead (1:3) o/n. The supernatant was collected at the end of activation and stored at −80 °C before protein analysis. The Luminex multiplex assay was performed using color-coded magnetic beads (Luminex, Austin, TX, USA) conjugated to antibodies against IL2, IL4, IL10, IL17α, TNF, IFNγ, and LAP (TGFβ), as described previously [23,24].

### 2.5. RNA-Sequencing

Total RNA was extracted using the RNAeasy kit (Qiagen, Hilden, Germany) according to the manufacturer’s protocol. mRNA was isolated using NEXTflex Poly(A) Beads (Bio Scientific, Austin, TX, USA), and libraries were prepared using NEXTflex Rapid Directional RNA-seq Kit (Bio Scientific). The quality of the RNA was assessed by the bioanalyzer on a pico-chip. Samples were sequenced using 75 bp single-end reads on an Illumina Nextseq 500 platform (Illumina Inc., San Diego, CA, USA) through the Utrecht sequencing facility (USEQ, Utrecht, The Netherlands). Reads aligned to the human genome (version GRCh37) and transcriptome (ENSEMBL version 37.74) with STAR version 2.4.2a were quantified at the gene level. Sample quality was evaluated as the number of expressed genes, and by Principal Component Analysis (PCA). Samples with fewer than 11,000 expressed genes were discarded. Samples were analyzed for differential gene expression with Voom-Limma using the eBayes functionality [25]. Gene set expression analysis was performed using CAMERA [26] with gene set information from MSigDB (version 6.2). In each case, TMMnormalized expression values were modeled using a linear model that included group treatment and donor information. Differential expression statistics were corrected for multiple testing by the Benjamini–Hochberg method [27]. Functional enrichment analysis of differentially expressed gene lists (adjusted *p*-value < 0.05) was detected using gProfiler2 0.2.3 [28] with Benjamini–Hochberg multiple testing correction. Gene Set Enrichment Analysis (GSEA) was conducted using fgsea package v1.30.0 [29].

### 2.6. Glucose Uptake Assay

Isolated CD4+ T-cells from 10 healthy donors stimulated o/n with aCD3/CD28 beads (1:3) in the presence of NAM9 mM versus control. For the final 10 min, cells were washed and incubated in glucose-free medium with 5% human AB-serum in the presence of 2-NBDG (glucose uptake assay kit ab235976 abcam, Cambridge, UK) in a concentration of 20 μg/mL and measured by flow cytometry.

### 2.7. ROS Measurement

Isolated CD4+ T-cells from 12 healthy donors stimulated o/n with aCD3/aCD28 beads (1:3) in the presence of nicotinamide 9 mM versus control. Cells were washed in PBS +2% FCS and subsequently stained in MitoSOX™ Red Mitochondrial Superoxide Indicator for live-cell imaging M36008 (BMDM) for 20 min and measured by flow cytometry.

### 2.8. Seahorse Assay

Isolated CD4+ T-cells were stimulated with anti-CD3 and anti-CD28 beads (1:3) for 24 h. Oxygen consumption rates (OCR) and extracellular acidification rates (ECAR) were measured in XF media (non-buffered RPMI 1640 containing 10 mM glucose, 2 mM L-glutamine, and 1 mM sodium pyruvate) under basal conditions and in response to glucose 30 mM, 1 μM oligomycin, and 50 mM of 2DG, on an XF-24 Extracellular Flux Analyzers (Seahorse Bioscience, Billerica, MA, USA).

### 2.9. Proliferation Assay

PBMCs were isolated from healthy donors as previously described and labeled in PBS with cell tracer violet (Life technologies, Carlsbad, CA, USA) in a final concentration of 2 μM for exactly 7 min at 37 °C and immediately washed in ice-cold 100% FCS once, followed by washing in ice-cold 10% FCS. Cells were subsequently incubated for 4 days in culture medium and stimulated with coated aCD3 1 μg/mL (OKT3 eBioscience^TM,^ San Diego, CA, USA) in the presence of NAM versus untreated control. Measurements were performed using the BD FACSCanto™ II flow cytometer, and FlowJo v10 was used for data analysis and calculating the division index. The division index represents the average number of cell divisions of the original cells, including the undivided cells.

### 2.10. Statistics

Statistical analysis was performed using GraphPad Prism 10. The statistical tests used to test significance are specified in the figure legends. *p*-values were calculated using a paired *t*-test or one-way ANOVA when indicated, ns: not significant, *: *p* < 0.05, **: *p* < 0.01, ***: *p* < 0.005, ****: *p* < 0.001.

## 3. Results

### 3.1. NAM Regulates T-Cell Activation Pathways

**Figure 1:**
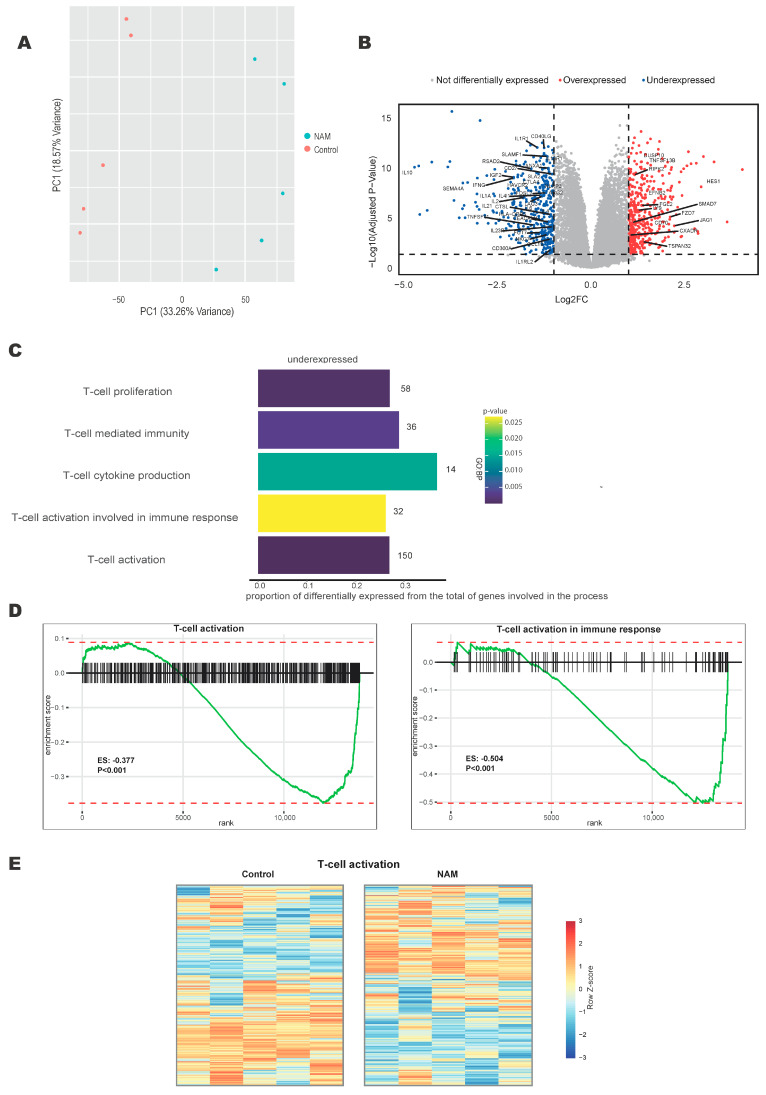
NAM regulates transcriptional pathways involved in T-cell activation. RNA sequencing performed on isolated primary human CD4+ T-cells stimulated with aCD3/CD28 beads in the presence of NAM versus control for 16 h. (N = 5). (**A**) Principal Component Analysis of gene expression data comparison for NAM treated and control samples. (**B**) Volcano plot comparing NAM treatment versus control. Genes significantly down- or upregulated (absolute log2FC > 1 and *p*-adjusted < 0.05) are presented by blue or red dots, respectively. The names of the significant genes involved in the enrichment of GO terms of C are shown. (**C**) Gene Ontology (GO-terms) of pathways associated with under-expressed genes after NAM treatment. (**D**) Gene Set Enrichment Analysis (GSEA) of the GO of Biological process (GO BP) functions; T-cell activation and T-cell activation involved in immune response. (**E**) Heatmap of differentially expressed genes in the GO geneset of T-cell activation in activated CD4+ T-cells in the presence of NAM versus untreated control. Expression determined by RNA sequencing. Colors indicate relative RNA expression on a scale from red (high) to blue (low).

To evaluate the effects of NAM treatment on CD4+ T-cell activation, isolated CD4+ T-cells from healthy donors were treated in vitro with or without co-incubation with NAM in the presence of TCR activation via aCD3/aCD28 beads for 16 h. Transcriptome-wide gene expression was analyzed through next-generation RNA sequencing. NAM treatment explained most of the variance between samples (Figure 1A) and resulted in 7674 differentially expressed genes (adjusted *p*-value < 0.05), of which 3880 were downregulated (Figure 1B). There was a significant over-presentation of downregulated genes associated with pathways involved in CD4+ T-cell activation and cytokine production (Figure 1C–E), indicating a regulatory effect of NAM on T-cell activation. T-cell viability was not affected by NAM treatment (Figure S1). Taken together, these data indicate that NAM reduces T-cell activation.

### 3.2. NAM Inhibits the Expression of T-Cell Surface Activation Markers

Gene expression profiling revealed distinct alterations in T-cell activation pathways following NAM treatment in vitro. Upon activation, T-cells upregulate various surface markers, including CD25 (IL-2R), HLA-DR (MHC class II), the co-inhibitory receptor CD279 (PD-1), and co-stimulatory molecules such as CD69 and CD40L [30]. These surface markers are expressed at different stages of T-cell activation; for example, CD69 is an early activation marker, while CD25 (a late marker) and HLA-DR (a very late marker) are upregulated at subsequent time points [31]. To assess the impact of NAM on CD4+ T-cell activation and function, the expression of these key surface markers was examined.

**Figure 2:**
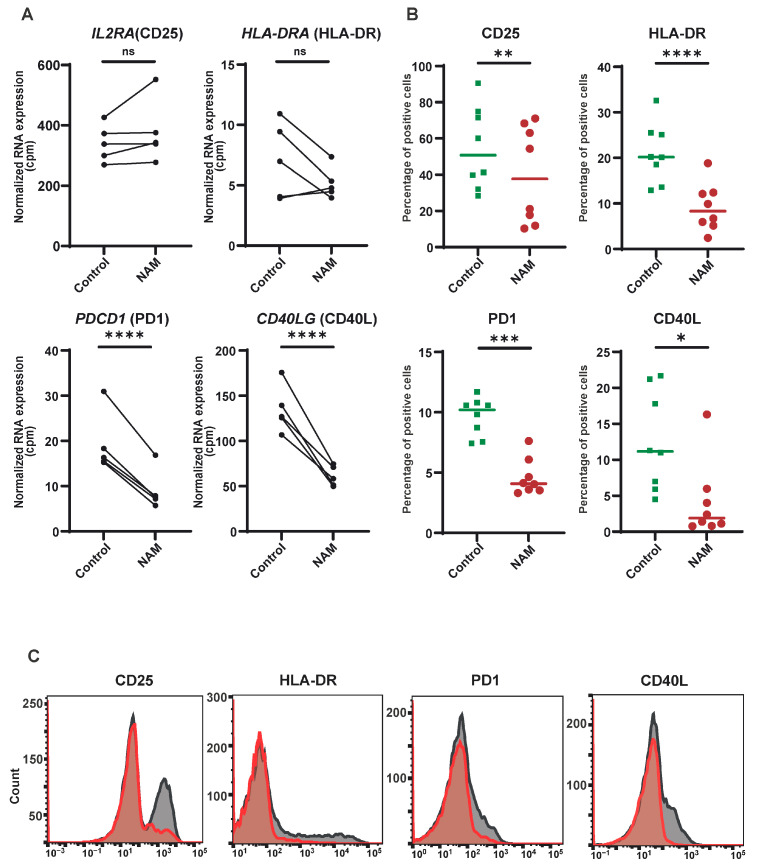
NAM inhibits T-cell activation displayed by reduction in surface activation markers. (**A**) Normalized RNA expression (CPM) of *IL2RA* (CD25), *PDCD1* (PD1), *HLA-DRA* and *CD40LG* (CD40L). (N = 5) (**B**) Isolated CD4+ T-cells stimulated with aCD3/aCD28 beads (1:20) in the presence of NAM versus control for 4 days and measured by flow cytometry. (N = 8) (**C**) Histograms of a representative example of B of untreated (gray) vs. NAM treated (red) CD4+ T-cells. *: *p* < 0.05, **: *p* < 0.01, ***: *p* < 0.005, ****: *p* < 0.001. ns: not significant.

RNA sequencing analysis revealed that NAM treatment resulted in reduced transcription of specific surface markers during the early activation stages (16 h, Figure 2A). To determine whether these changes in expression were also reflected in protein level, the surface expression of CD25, PD1, HLA-DR, and CD40L was measured in CD4+ T-cells that were treated with NAM for four days (Figure 2B,C). At the protein level, NAM had a more pronounced effect, reducing the protein expression of these four activation-associated surface markers. While CD69 surface expression remained unchanged at both transcriptional and protein levels four days after activation, a reduction in CD69 surface expression was observed as early as four hours post-activation with NAM pre-treatment, as confirmed by flow cytometry (Figure S3A–D).

Collectively, these data demonstrate that NAM suppresses T-cell activation as measured by surface marker expression. CD69 and PD-1 also serve regulatory roles in immune responses; therefore, the consequence of attenuating these surface markers in combination with other cell surface markers warrants further investigation to understand the effects on T-cell proliferation and function [32,33,34].

### 3.3. NAM Inhibits CD4+ T-Cell Proliferation

**Figure 3:**
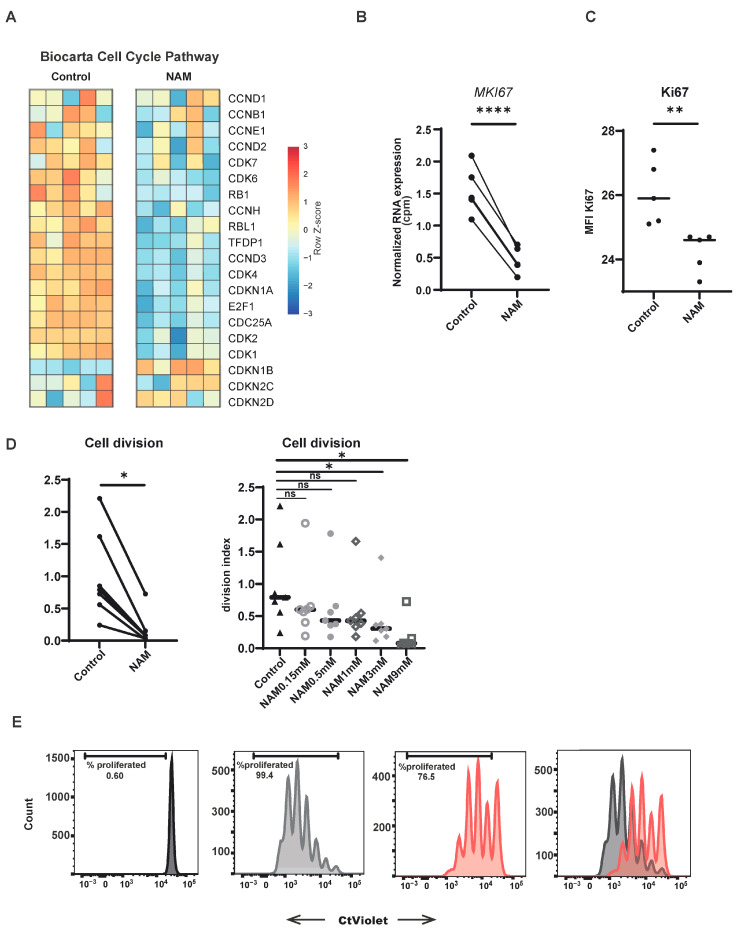
NAM inhibits CD4+ T-cell proliferation. (**A**) Heatmap of differentially expressed genes in the Biocarta geneset of cell division in activated CD4+ T-cells in the presence of NAM versus untreated control. Expression determined by RNA sequencing. Colors indicate relative RNA expression on a scale from red (high) to blue (low). (**B**) Normalized RNA expression (CPM) of *MKI67* (Ki67) (N = 5). (**C**) MFI of Ki67 expression measured on flow cytometry in CD4+ T-cells (N = 5). (**D**) PBMCs labeled with ctViolet and stimulated with coated aCD3 for 4 days in the presence of different concentrations of NAM (0.15 mM/0.5 mM/1 mM/3 mM/9 mM) versus untreated control. Division index of CD4+ T-cells, selected by gating on CD3 + CD4+ (gating strategy S2). Statistical significance was measured by one-way ANOVA. (N = 7) (**E**) Representative example of proliferation shown in D of unstimulated (black) untreated (gray) versus NAM treated (red) CD4+ T-cells. *: *p* < 0.05, **: *p* < 0.01, ****: *p* < 0.001, ns: not significant.

An essential aspect of T-cell function is its capacity to proliferate following antigen recognition and receptor binding. The observed reduction in T-cell activation in the presence of NAM was concomitant with a downregulation of the transcriptional expression of genes involved in cell cycle pathways following NAM treatment (Figure 3A). To validate these observations, freshly isolated human T-cells were activated and treated with NAM, and proliferation was measured by the expression of Ki67, a well-established marker of cell proliferation. Indeed, NAM treatment reduced the expression of *MKi67* (Ki67) and Ki67 protein in these cells (Figure 3B,C). To further quantify the impact of NAM on T-cell proliferation, peripheral blood mononuclear cells (PBMCs) from healthy donors were labeled with Celltrace Violet and subsequently activated in the presence of NAM. After four days, NAM-treated cells exhibited a reduction in cell division, as indicated by a lower division index (Figure 3D). Notably, the inhibitory effect of NAM on proliferation was observed to be dose-dependent and was not restricted to CD4+ T-cells only, as it was also demonstrated in CD8+ T-cells (Figure 3C and Figure S6A).

### 3.4. NAM Reduces Pro-Inflammatory Cytokine Production in TCR Activated CD4+ T-Cells

**Figure 4:**
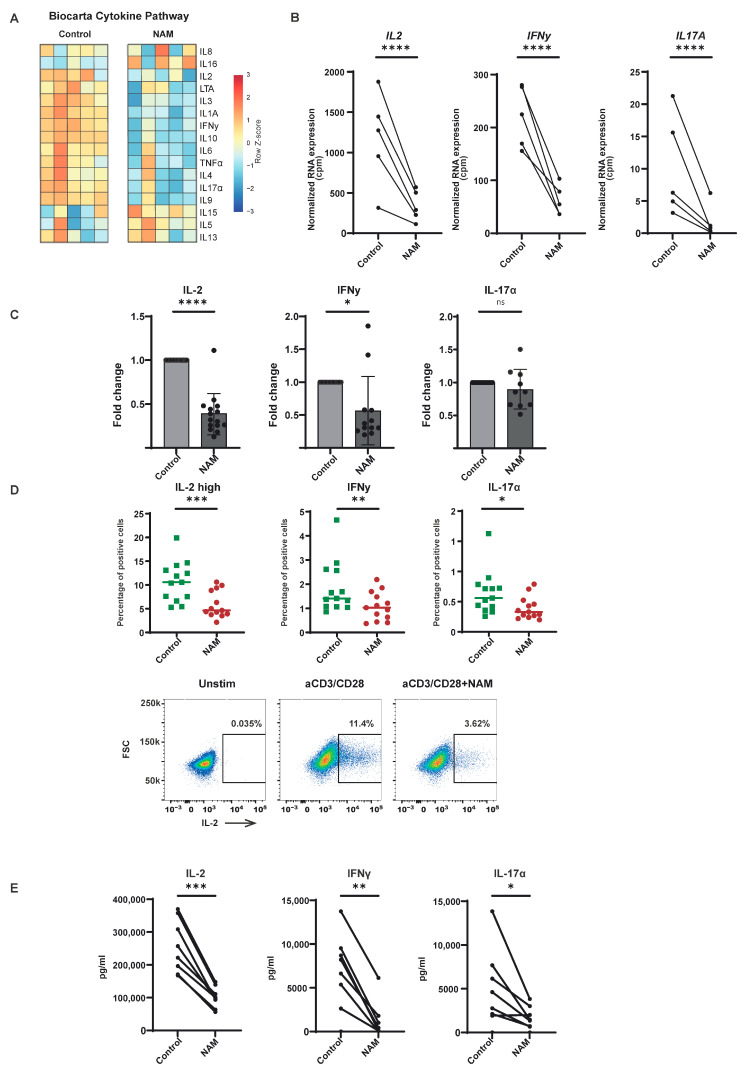
NAM suppresses the production of pro-inflammatory cytokines. (**A**) Heatmap of differentially expressed genes in the Biocarta geneset of pro-inflammatory cytokines in activated CD4+ T-cells in the presence of NAM versus control. Expression determined by RNA-sequencing. Colors indicate relative RNA expression on a scale from red (high) to blue (low). (**B**) Normalized RNA expression (CPM) of *IL2/IFNG* and *IL17A*. (N = 5) (**C**) Relative mRNA expression of IL-2/IFNy and IL-17α to its own internal control (set to 1) in isolated activated CD4+ T-cells and measured by qPCR after incubation with NAM for 18 h versus untreated control. (N = 15 (IL-2), N = 12 (IFNy), N = 10 (IL-17α)) (**D**) Intracellular protein expression of IL-2 high/IFNγ and IL-17α of isolated activated CD4+ T-cells after incubation with NAM for 18 h versus untreated control and measured by flow cytometry. (N = 13) (**E**) Quantitative analysis of IL-2, IFNγ, and IL-17α in the supernatant of isolated activated CD4+ T-cells after incubation with NAM for 18 h versus untreated control and measured by Luminex. (N = 8) *: *p* < 0.05, **: *p* < 0.01, ***: *p* < 0.005, ****: *p* < 0.001, ns: not significant.

Given the observed suppression of CD4+ T-cell activation, as evidenced by reduced expression of surface activation markers and cell proliferation, the effect of NAM on cytokine production was evaluated. NAM treatment resulted in the downregulation of the expression of genes involved in the cytokine pathway (Figure 4A,B and Figure S4A). This was confirmed by measuring mRNA levels of Interleukin-2 (IL-2), Interferon-gamma (IFNy), Tumornecrosefactor-α (TNFα), and Interleukin-17 (IL-17α) with qPCR in isolated activated CD4+ T-cells in the presence of NAM (NS for IL-17α) (Figure 4C and Figure S4B). To obtain a more direct readout of the actual protein expression of these cytokines, both flow cytometry and Luminex assay were used (Figure 4D,E and Figure S4C). A decreased protein expression and excretion of several important pro-inflammatory cytokines were observed, including but not limited to IL-17α, IL-2 high, and IFNy (Figure 4E and Figure S5). Notably, this effect was not confined to CD4+ T-cells, as a similar inhibition of TNFα production was observed in CD8+ T-cells (Figure S6B,C). In conclusion, NAM treatment reduces the expression of different pro-inflammatory cytokines in CD4+ T-cells both on mRNA and protein levels.

### 3.5. NAM Impairs Metabolic Processes Essential for T-Cell Activation

**Figure 5:**
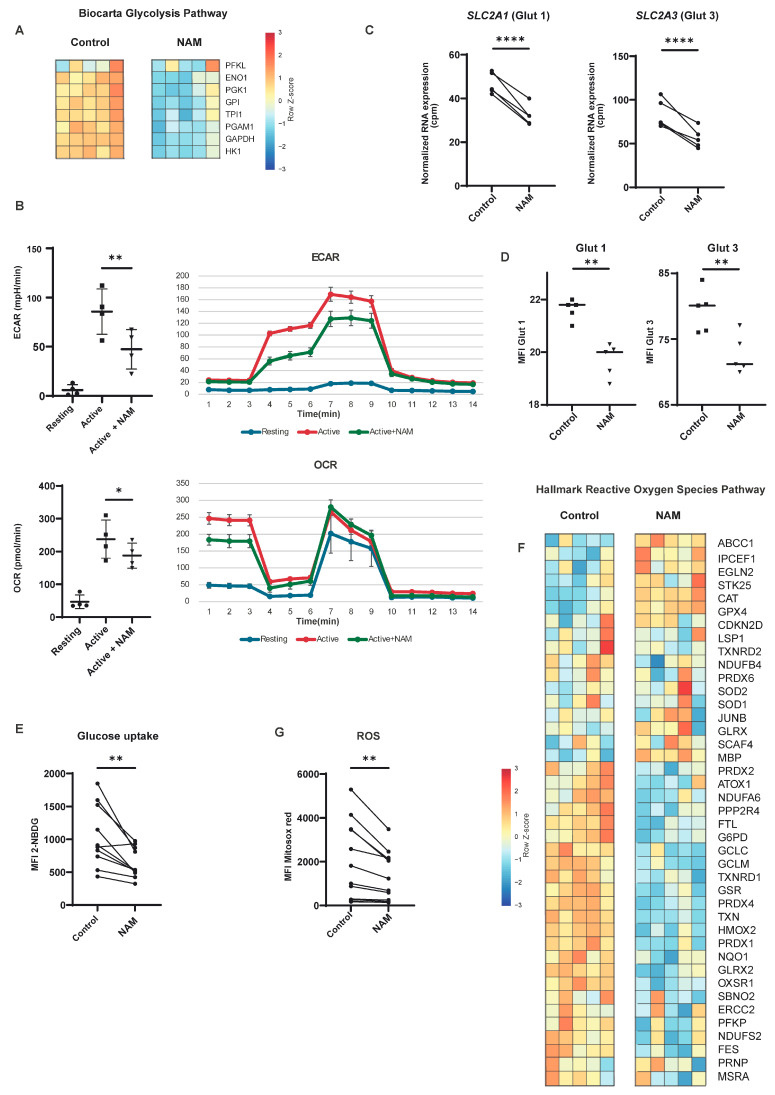
NAM leads to a reduction in key metabolic processes in T-cell activation. (**A**) Heatmap of differentially expressed genes in the Biocarta geneset of glycolysis in activated CD4+ T-cells in the presence of NAM versus control. Expression determined by RNA-sequencing. Colors indicate relative RNA expression on a scale from red (high) to blue (low). (**B**) Oxygen consumption rate (OCR) and extracellular acidification rate (ECAR) were measured in activated CD4+ T-cells in the absence or presence of NAM. (N = 4) (**C**) Normalized RNA expression (CPM) of *SLC2A1* (Glut 1) and *SLC2A3* (Glut3). Expression determined by RNA-sequencing. (N = 5) (**D**) MFI of Glut1 and Glut3 in activated CD4+ T-cells in the presence of NAM versus control. (N = 5) (**E**) Glucose uptake assay. MFI of 2-NBDG in activated CD4+ T-cells in the presence of NAM versus control. (N = 10) (**F**) Heatmap of differentially expressed genes involved in reactive oxygen species (ROS) production of activated CD4+ T-cells in the presence of NAM versus control. Expression determined by RNA-sequencing. Colors indicate relative RNA expression on a scale from red (high) to blue (low). (**G**) ROS production. MFI of Mitosox red in activated CD4+ T-cells in the presence of NAM versus control. (N = 10) *: *p* < 0.05, **: *p* < 0.01, ****: *p* < 0.001.

Resting T-cells primarily rely on oxidative phosphorylation (OXPHOS) to meet their energy demands. Upon activation of T-cells via the T-cell receptor, there is a marked increase in metabolic demands, which is, amongst other things, reflected by a metabolic reprogramming, including enhanced aerobic glycolysis, increased glucose uptake through upregulation of cell-surface glucose transporters and a rise in OXPHOS. This increase in OXPHOS contributes to the production of reactive oxygen species (ROS), which in turn triggers key signaling pathways essential for T-cell activation [35,36,37,38]. Therefore, we investigated the effect of NAM on glycolysis, glucose uptake, and ROS production. NAM treatment during TCR activation in vitro resulted in a downregulation of genes involved in glycolysis (Figure 5A). To validate that NAM could functionally impair glycolysis after activation, the oxygen consumption rate (OCR) and extracellular acidification rate (ECAR), both key indicators of glycolytic activity, were measured by Seahorse metabolic analysis (Figure 5B). Indeed, a significant reduction was observed. For glycolysis to proceed efficiently, a sufficient supply of glucose must be available in the cell, making adequate glucose uptake essential. In our study, we observed a reduced expression of glucose transporters Glut1 and Glut 3, as well as a decrease in glucose uptake in NAM-treated CD4+ T-cells (Figure 5C–E). This impairment in glucose uptake likely contributes to the observed decrease in glycolytic activity. Next to reduced glycolytic activity, a decreased T-cell activation status would be expected to be reflected in decreased ROS production. As expected, NAM treatment resulted in a down-regulation of genes involved in ROS production (Figure 5F). In addition, we observed a significant reduction in intracellular ROS as a result of NAM treatment (Figure 5G). These findings suggest that NAM impacts core metabolic pathways critical for T-cell activation and function.

## 4. Discussion

CD4 + T-cells play a crucial role in the pathogenesis of autoimmune diseases. Therefore, the inhibition or regulation of CD4+ T-cells could be a promising target for therapeutic interventions. NAM has demonstrated considerable potential in the treatment of such conditions by modulating the activation of different immune cells [15,21,22,39]; however, its precise effects on CD4+ T-cell function remain largely unknown. Here, we demonstrate that NAM can directly impair CD4+ T-cell activation, reflected in a reduction in surface activation markers, cellular proliferation, and pro-inflammatory cytokine production. Taken together, our study provides novel insights into the molecular mechanisms that control T-cell activation and proposes NAM treatment as a promising and relatively safe therapeutic candidate to suppress immune activation.

Although the underlying mechanisms through which NAM exerts these immune modulatory effects remain to be fully elucidated, several potential pathways have been proposed, reflecting the complexity of its action. NAM is a well-documented inhibitor of PARP-1 and the NAD+-dependent deacetylase SIRT-1, both of which are key regulators of different cellular functions, including immune responses [40,41]. The impact of NAM on SIRT-1 activity remains a subject of ongoing debate. While NAM is a direct inhibitor of SIRT-in vitro, NAM is also a precursor of NAD+ via its conversion to NAD+ through the salvage pathway, a process mediated by NAMPT, making it essential for SIRT-1 function. Consequently, the impact of NAM on SIRT-1 activity and subsequent immune modulation is complex and may vary depending on the cellular context [42]. Furthermore, NAD+ itself is critical for various cellular processes, including energy metabolism, DNA repair, and immune cell function [43,44]. Therefore, NAM’s influence on immune cells can be multifaceted, extending beyond the modulation of specific enzymes like SIRT-1 and PARP-1. In CD8+ T-cells, for example, NAM’s inhibitory effects have been shown to occur independently of SIRT-1 and involve the mTOR signaling pathway [21]. Further studies involving the reversal of the effect of NAM through inhibition of SIRT-1 and PARP-1, along with blocking the conversion of NAM to NAD+ using NAMPT inhibition, could elucidate the underlying mechanisms and clarify the distinct pathways through which NAM exerts its effects in T-cells.

It is important to note that this study primarily focuses on the effect of NAM on CD4+ T-cells in a broad context. However, it is well known that CD4+ T-cells are a heterogeneous population consisting of various subsets, each with distinct roles in immune regulation in both health and disease. Therefore, it would be valuable to assess the effects of NAM on specific CD4+ T-cell subsets, as well as on CD4+ T-cell differentiation pathways. This could provide deeper insight into how NAM influences CD4+ T-cell plasticity and function and their potential to differentiate into more regulatory or pro-inflammatory subtypes.

Most studies investigating the in vivo effect of NAM show an immunoregulatory role for NAM [9,11,12]. However, the effect of NAM can vary depending on the biological context and the specific cell types involved. For example, research on non-melanoma skin cancer (NMSC) hypothesizes a beneficial role of NAM in preventing NMSC and demonstrates the effectiveness of oral NAM suppletion in reducing UV-induced immunosuppression [45,46]. There was a dose-dependent reduction in immunosuppression observed in NAM-treated individuals. One of the proposed mechanisms of NAM’s effect is enhanced DNA repair via NAD+ and PARP-1 inhibition. Although the in vivo impact of NAM on the immune (particularly T-cell) function requires further investigation, an important consideration in exploring the therapeutic potential of NAM in autoimmune diseases is its safety profile. Numerous clinical trials involving large groups of participants have demonstrated that NAM is safe for long-term use. In these studies, high doses of up to 3 g a day of NAM were administered over periods of up to five years, with participants involving both adults and children from the age of five. Throughout all these trials, no serious adverse events were reported, and treatment was never discontinued due to side effects [9,12,47,48,49,50]. This further supports its potential as a therapeutic strategy for managing autoimmune disorders.

In conclusion, the findings of our study indicate that NAM modulates critical aspects of T-cell function. Given the safety of long-term use and anti-inflammatory effects on CD4+ and CD8+ T-cells, we propose NAM as a promising therapeutic approach, specifically as an add-on medication or as a maintenance therapy in relapsing-remitting diseases like RA or JIA. Additional studies addressing the exact molecular mechanisms underlying NAM’s effect on CD4+ T-cells, along with in vivo confirmation of these effects, could strengthen the foundation for advancing NAM as a potential treatment for immune-related diseases.

## Data Availability

The original contributions presented in this study are included in the article/Supplementary Materials. Further inquiries can be directed to the corresponding author.

## Supplementary Materials

The following supporting information can be downloaded at: https://www.mdpi.com/article/10.3390/cells14080560/s1, Figure S1: Effect of NAM on cell survival, Figure S2: Gating strategy used in Figure 2, Figure S3: Effect of NAM on CD69 expression, Figure S4: Effect of NAM on TNF expression, Figure S5: Supplemental quantitative analysis of Il-4, IL-10,IL-13, TGF-β (LAP), Figure S6: NAM inhibits proliferation and cytokine production in CD8+ T-cells, Table S1: List of antibodies used for flow cytometry.
