# Supplementary material for: Nicotinamide Inhibits CD4+ T-Cell Activation and Function
Source: Cells. 2025 Apr 8;14(8):560. doi: 10.3390/cells14080560 (PMC12025565; doi:10.3390/cells14080560)
Supplement: Supplementary file 1 [file cells-14-00560-s001.zip › Figure legends supplemental figures.pdf]

**Figure S1**. Effect of NAM in increasing dosages on cell survival. Percentage of dead cells detected by fixable viability dye (eF506) after overnight activation and incubation with NAM (0.3mM/1mM/3mM/9mM) versus untreated control. Statistical significance was determined by one-way ANOVA. (N=4)
ns: not significant.

**Figure S2.** Gating strategy Figure 2B

**Figure S3.** Effect of NAM treatment on CD69 expression**. (A)** Normalized RNA expression (CPM) of *CD69*. Expression determined by RNA-sequencing. (N=5) **(B)** Isolated CD4+ T-cells stimulated with aCD3/CD28 beads (1:20) in the presence of NAM versus control for 4 days and measured by flow cytometry. (N=8) **(C)** Isolated CD4+ T-cells stimulated with aCD3/aCD28 beads for 4 hours after 1 hour of pre-incubation with NAM and measured by qPCR. (N=7) **(D)** Isolated CD4+ T-cells stimulated with aCD3/aCD28 beads for 4 hours after 1 hour of pre-incubation with NAM and measured by flow cytometry. (N=5)

**: $p < 0.01$, ns: not significant

**Figure S4.** Effect of NAM treatment on TNF$\alpha$ expression. **(A)** Normalized RNA expression (CPM) of *TNF*. Expression determined by RNA- sequencing. (N=5) **(B)** Relative mRNA expression of TNF$\alpha$ in isolated activated CD4+ T-cells and measured by qPCR after incubation with NAM for 18 hours versus untreated control. (N=8) **(C)** Intracellular protein expression of TNF$\alpha$ of isolated activated CD4+ T-cells after incubation with NAM for 18 hours versus untreated control and measured by flow cytometry (non-significant). (N=9)
*: $p < 0.05$, **: $p < 0.01$, ***: $p < 0.005$, ****: $p < 0.001$, ns: not significant

**Figure S5.** Supplemental quantitative analysis of IL-4, IL-10, IL-13, TGF-β (LAP) in supernatant of isolated activated CD4+ T-cells after incubation with NAM for 18 hours versus untreated control and measured by Luminex. (N=8)
**: $p < 0.01$, ***: $p < 0.005$, ns: not significant

**Figure S6.** NAM inhibits proliferation and cytokine production in CD8+ T-cells**. (A)** PBMCs labelled with ctViolet and stimulated with coated aCD3 for 4 days in the presence of different concentrations of NAM (0.15mM/0.5mM/1mM/3mM/9mM) versus untreated control. Division index of CD8+ T-cells, selected by gating on CD3+CD8+. Statistical significance was measured by one way ANOVA. (N=7) **(B)** Relative mRNA expression of TNF$\alpha$ in isolated activated CD8+ T-cells and measured by qPCR after incubation with NAM for 18 hours versus untreated control. (N=8) **(C)** Intracellular protein expression of TNF$\alpha$ of isolated activated CD8+ T-cells after incubation with NAM for 18hours versus untreated control and measured by flow cytometry. (N=6)
*: $p < 0.05$, **: $p < 0.01$, ***: $p < 0.005$, ns: not significant
