# Supplementary material for: Nicotinamide Inhibits CD4+ T-Cell Activation and Function
Source: Cells. 2025 Apr 8;14(8):560. doi: 10.3390/cells14080560 (PMC12025565; doi:10.3390/cells14080560)
Supplement: Supplementary file 1 [file cells-14-00560-s001.zip › Supplementary table S1.pdf]

Supplementary Table 1; List of antibodies used for flow cytometry

Marker	Fluochrome	Catalog no	Company
Fixable Viability Dye	eFluor 506	15560607	eBioscience
Mouse anti-CD3	PerCp-Cy5.5	300430	Biolegend
Mouse anti-CD4	APC	17-0049-42	eBioscience
Mouse anti-IFNy	PE-Cy7	557844	BD Biosciences
Rat anti-IL2	Pacific Blue	500324	Biolegend
Mouse anti -IL17α	PE	12-7179-42	eBioscience
Mouse anti- CD69	FITC	130-113-523	Miltenyi Biotec
Mouse anti- CD279 (PD-1)	PerCp-Cy5.5	329914	Biolegend
Mouse anti-CD25 (IL2RA)	PE-Cy7	557741	BD Biosciences
Mouse anti-CD154 (CD40L)	APC	17-1548-42	eBioscience
Mouse anti-HLA-DR (MHCII)	Pacific Blue	307624	Biolegend
Mouse anti-CD4	PE	300508	Biolegend
Mouse anti-CD3	PerCp-cy5.5	300430	Biolegend
Mouse anti-CD8	FITC	555366	BD Biosciences
Celltracer Violet	Violet 1	C34557	Life Technologies
Mouse anti-CD3	PerCp-cy5.5	300430	Biolegend
Mouse anti-CD8	FITC	555366	BD Biosciences
Celltracer Violet	Violet 1	C34557	Life Technologies
Fixable Viability dye	eFluor 780	65-0865-14	eBioscience
Mouse anti-Glut 1	AF647	566580	BD Biosciences
Rabbit anti-Glut 3	FITC	GLUT3-FITC	Life technologies
Mouse anti-Ki67	AF647	558615	BD Biosciences
