# Supplementary figures and images for: Nicotinamide Inhibits CD4+ T-Cell Activation and Function
Source: Cells. 2025 Apr 8;14(8):560. doi: 10.3390/cells14080560 (PMC12025565; doi:10.3390/cells14080560)

S1

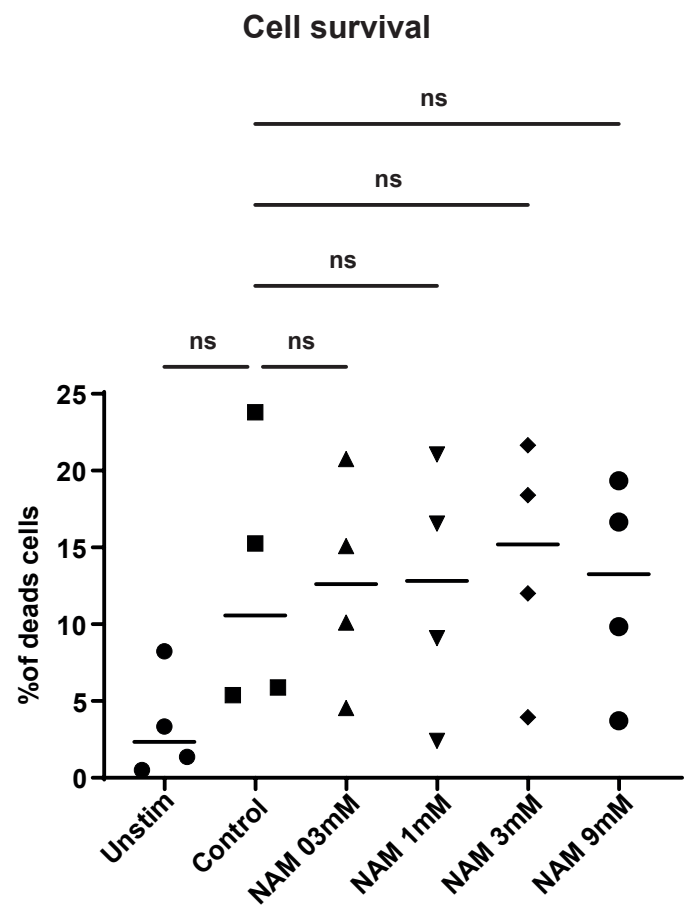

Supplement: Supplementary file 1 [file cells-14-00560-s001.zip › Figure S1.pdf]

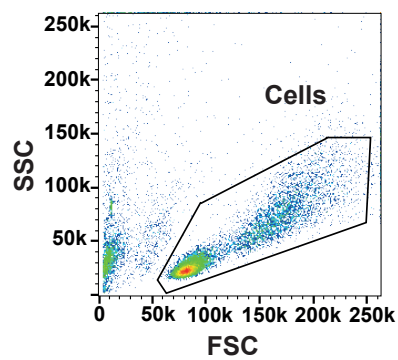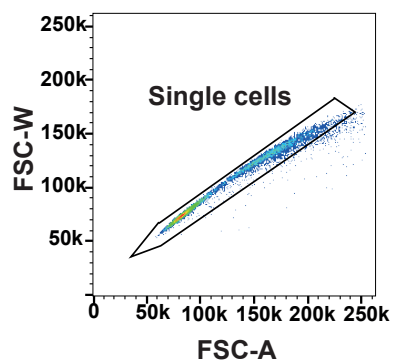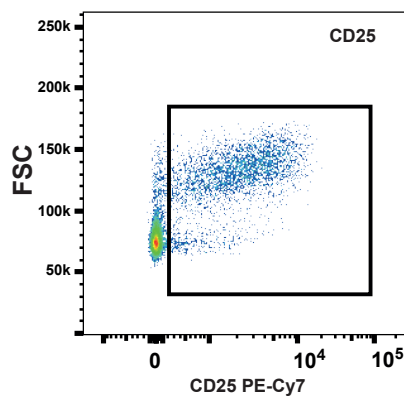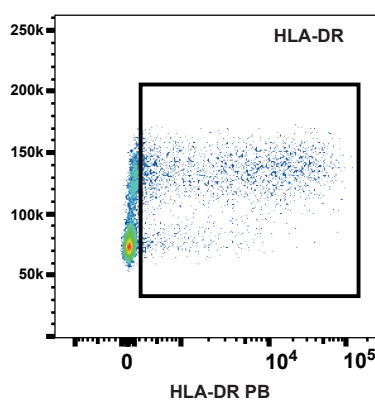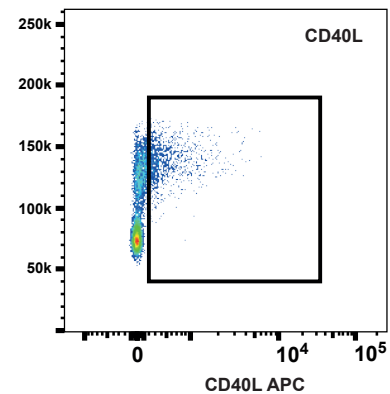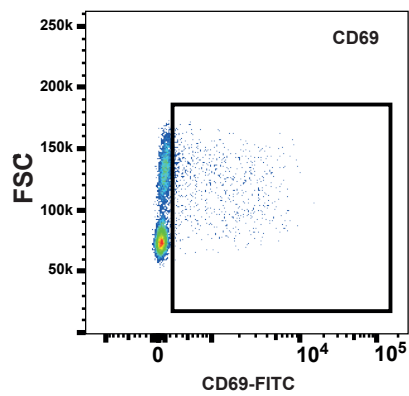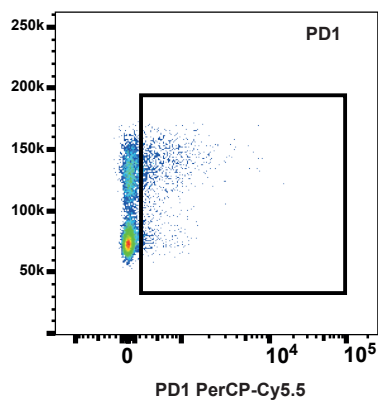

Supplement: Supplementary file 1 [file cells-14-00560-s001.zip › Figure S2.pdf]

**A**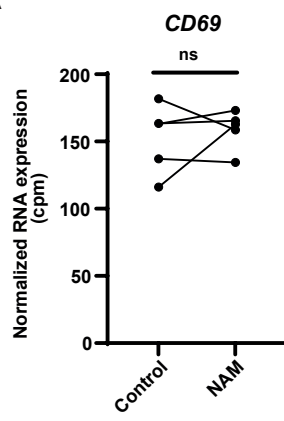**B**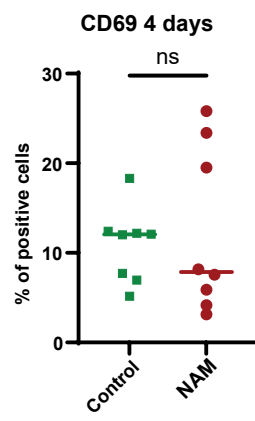**C**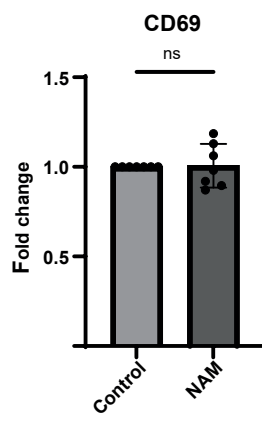**D**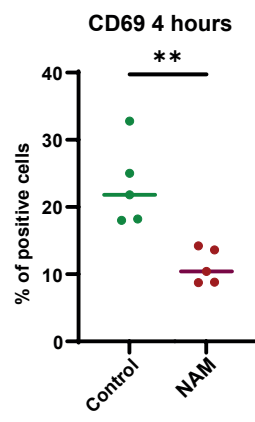

Supplement: Supplementary file 1 [file cells-14-00560-s001.zip › Figure S3.pdf]

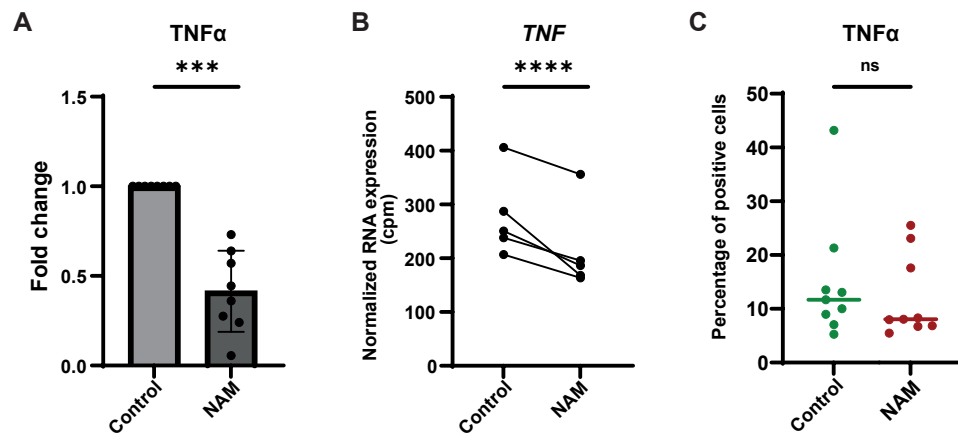

Supplement: Supplementary file 1 [file cells-14-00560-s001.zip › Figure S4.pdf]

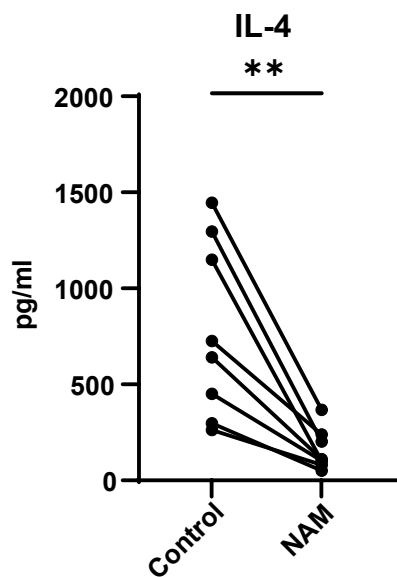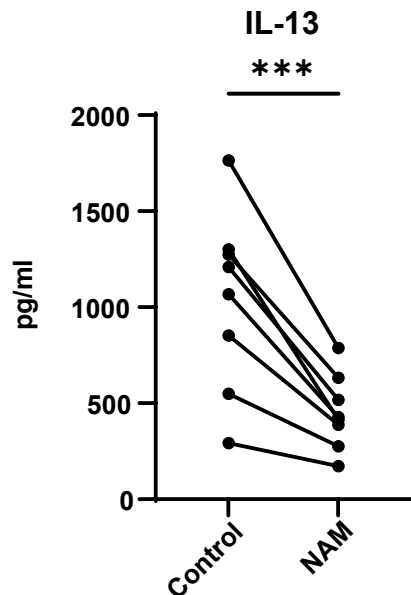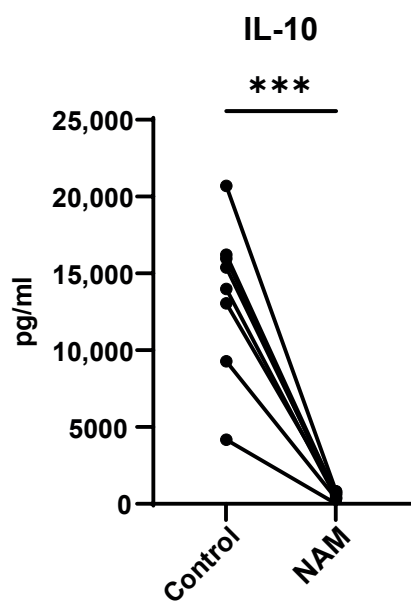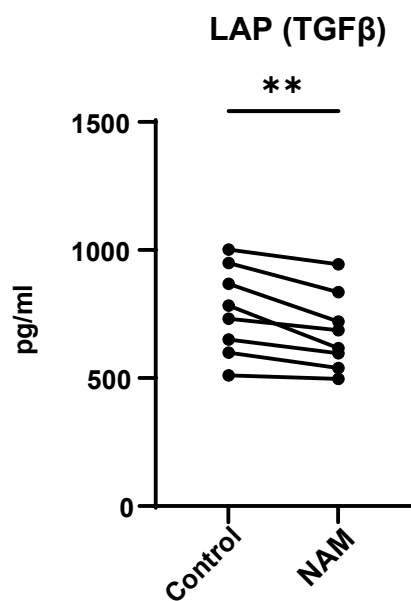

Supplement: Supplementary file 1 [file cells-14-00560-s001.zip › Figure S5.pdf]

A

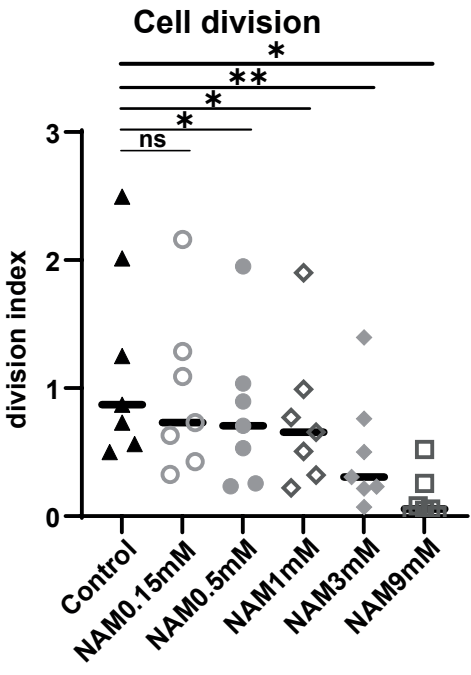

B

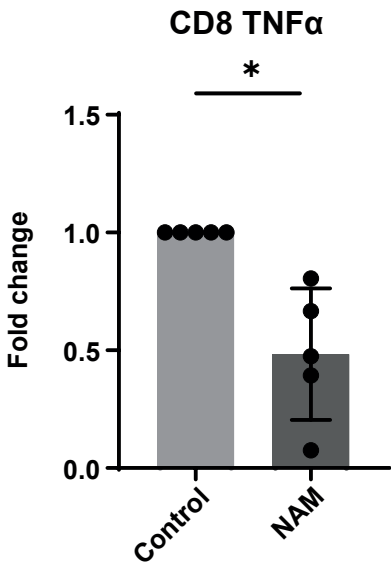

C

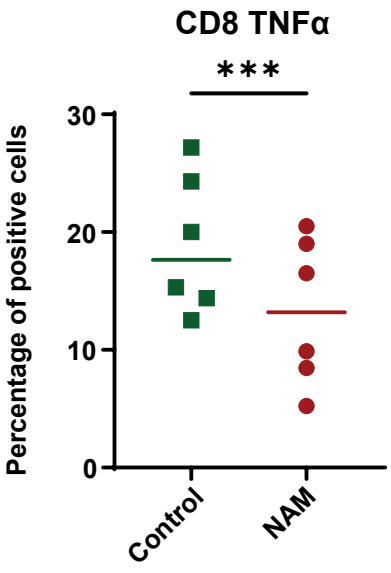

Supplement: Supplementary file 1 [file cells-14-00560-s001.zip › Figure S6.pdf]
